# Clinical Evaluation on the Performance and Safety of a Non-Ablative Fractional 1340 nm Laser for the Treatment of Stretch Marks in Adolescents and Young Adults: A Case Series

**DOI:** 10.3390/bioengineering9040139

**Published:** 2022-03-25

**Authors:** Maria Teresa Viviano, Alessia Provini, Cinzia Mazzanti, Steven Paul Nisticò, Cataldo Patruno, Giovanni Cannarozzo, Stefano Bennardo, Irene Fusco, Luigi Bennardo

**Affiliations:** 1 Ospedale San Pietro-Fatebenefratelli, 00100 Rome, Italy; mt.viviano@gmail.com (M.T.V.); c.mazzantiderm@gmail.com (C.M.); 2 Istituto Dermopatico dell’Immacolata, 00100 Rome, Italy; alessiaprovini@yahoo.it; 3 Department of Health Sciences, Magna Graecia University, 88100 Catanzaro, Italy; nistico@unicz.it (S.P.N.); cataldopatruno@libero.it (C.P.); drcannarozzo@gmail.com (G.C.); stefano.bennardo@studenti.unicz.it (S.B.); luigi.bennardo@studenti.unicz.it (L.B.); 4 Department of Pharmacology, Università degli Studi di Firenze, 50100 Florence, Italy

**Keywords:** striae, stretch marks, non-ablative fractional laser

## Abstract

A large part of the world’s population suffers from Striae distensae (SD) or stretch marks, which create physical and psychological discomfort in people. We evaluate the SD clinical improvement by using a non-ablative fractional Nd:YAP 1340 nm laser. The research was performed on 25 patients of both sexes, with a mean age of 31 ± 13.09 years. Each patient underwent from a minimum of 3 to a maximum of 4 treatments, with an Nd:YAP (1340 nm) medical device, every four weeks, with 3- and 6-month follow-up, in these areas: back, abdomen, breast, flanks, lower limbs, buttocks, and thighs. Manchester Scar Scale assessed stretch marks improvement. Side effects, patient pain, and SD overall appearance improvement were also recorded for all patients. Digital photographs measured the aesthetic results. Treatment was well-tolerated (pain score 1.08 ± 0.76) by all patients. There were no long-term side effects, and 88% of patients revealed an SD excellent improvement showing good aesthetic results achieved by the treatment. The total mean pretreatment Manchester Scar Scale score decreased from 13.80 (±1.58) to 10.36 (±1.70) after 3 months (*p* < 0.01) and to 8.36 (±1.07) after 6 months (*p* < 0.01). An Nd:YAP (1340 nm) laser seems to be a safe and effective treatment, showing a higher security profile with no side effects.

## 1. Introduction

Striae distensae (SD), or stretch marks, are prevalent skin lesions with linear features of atrophic skin [1]. To date, these skin scars characterized by epidermal atrophy are of great interest in the aesthetic field [2,3]. In SD, there is a rapid elongation of the skin, which causes a reorganization of reticular collagen [4]. At the epidermal level, there is a loss of dermal papillae and the formation of network crests, while the dermis shows a de-crease in the components of the extracellular matrix (ECM); fibronectin, fibrillin, collagen, and elastin [5,6]. The dermis consists of a matrix of elastin and collagen [7]. In healthy skin, it is possible to observe that collagen fibrils, organized in densely packed bundles [8], permit the skin to stretch and then return to its native shape [9,10,11]. The collagen bundles are separated following SD development, not allowing the collagen fibrils to reform the bundles. Following the destruction of the elastic fibers, fibrils rich in tropoelastin (soluble elastin) can no longer organize themselves into normal elastic fibers [8]. Stretch marks are caused by the breakdown of elastic fibers due to excessive mechanical stretching of the skin and the inability of local fibroblasts to repair or re-place ECM components [6]. They are common in pregnant women and adolescents. Among adolescents, the prevalence of SD ranges from 6% to 86%. Male adolescents are most commonly affected in the lower back and knees, while females are most frequently affected in the calves, buttocks, and thighs. SD can generally develop for three reasons: hormonal changes, mechanical stretching of the skin, or innate structural disorders [12]. SD is hypothesized to result from an inflammatory reaction triggered at the beginning that can destroy elastic fibers and collagen, which are regenerated in the direction imposed by mechanical forces [13]. When SD develops for the first time, the vascular component is affected, creating edematous scars (pink-red-purple) called stria rubrae (SR). There is a decrease in vascularization over time, leading to local degradation of collagen and elastin with stria albae (SA) formation, characterized by an atrophic appearance [14,15]. Pierard–Franchimont et al. [16] observed that as melanization increases, particularly in patients with dark skin, two different types of striae are created (striae caerulea and striae nigrae). During adolescence, physiological striae appear around puberty, mainly in healthy and non-obese individuals [17]. There are no treatments for all types of existing SD on the market, also relating to various skin types [18,19]. Available treatments include chemical peels, topical agents, microdermabrasion, and ablative and non-ablative energy devices [19]. Various non-ablative or ablative laser techniques are used for the treatment of SD: pulsed dye laser (PDL) [20], using a 595 nm wavelength to selectively hit scar vascularization [21,22,23] excimer laser [24], 1064 nm neodymium-doped YAG (Nd-YAG) laser [25], with its ability to target dermis sparing the superficial skin layers [26,27], 577 nm copper bromide laser [28], non-ablative fractional laser [29,30,31,32] and CO_2_ laser [33] (although the latter has a hyperpigmentation risk for dark skin types [34,35]). The most recent applications have introduced the Fractional photothermolysis (FP), which produces microscopic treatment zones (MTZs) to stimulate a rapid wound healing response. The US FDA recently ap-proved this 1540 nm wavelength modality for the treatment of SA [36]. These microscopic columns are surrounded by unheated tissue, which causes keratinocytes migration into damaged areas to induce rapid healing [37,38]. In using non-ablative lasers, epidermis protection is achieved by using a cooling system that ameliorates patient tolerability compared to ablative lasers. According to the literature, there is evidence of good clinical and histopathological results for the abdominal SA treatment with NAFL Nd: YAP 1340 nm and Microneedling [39]. With deep penetration targeting water within the cells, the 1340 nm infrared wavelength provides greater efficacy with a shorter recovery time than traditional 1540 nm lasers. This research aimed to assess the performance of the non-ablative Nd: YAP 1340 nm fractional laser in stretch marks reduction.

## 2. Materials and Methods

### 2.1. Patient Selection

In this study, 25 patients (17 females and eight males) with a mean age of 31 ± 13.09 years and affected by Striae distensae, were selected and enrolled at a private clinic. According to Fitzpatrick’s classification, the phototype of the patients ranged from type II to type III. All patients were adequately informed on the procedure’s treatment and possible side effects. All the patients underwent, before and after treatment, photographic evaluation. Patients were re-evaluated three months and six months after the last treatment for one better evaluation of the results obtained in the long term, whereas all the biostimulation methods of the collagen take at least three months to achieve the desired effect.

The exclusion criteria included: hypersensitivity to light; intake of photosensitizing drugs, anticoagulant drugs, medications that alter the regular tissue repair, isotretinoin for at least six months; application of isotretinoin or other substances sensitizers on the area to be treated, which must have been suspended for at least one month; recent sun exposure; bacterial, viral or fungal infections in place.

Before the study began, all participants provided their written, informed consent. Moreover, all patients agreed to use their photographs for scientific purposes.

### 2.2. Pre-Treatment Procedures

Before proceeding with the treatment, all the areas were cleaned using a mild soap and rinsed with water. All patients were orally administered one tablet of Piascledine (300 mg) and one tablet of Vitamin E daily for the entire treatment period.

### 2.3. Study Protocol

The objective of this clinical study was to evaluate the clinical performance of Nd:YAP 1340 nm laser (Insight Handpiece, Luxea, DEKA M.E.L.A., Calenzano, Italy) treatment on non-invasive stretch marks (Striae distensae) reduction on the back area, abdomen, flanks, buttocks, lower limbs, breast and thighs. The system was equipped with 1 handpiece with a weight of 400 g (200 × 70 × 30 mm) and an integrated cooling system (5–25 °C). In this clinical study, 3/4 treatment sessions every four weeks were performed, on each area, for a length of 20 min per area, according to the following settings: 1 Pass- Fluence 12–18 J/cm^2^-400 DOT/cm^2^. The treatment involves the passage of the handpiece in contact with the skin surface to be treated, without applying excessive pressure, with spots that follow one another without overlap but without leaving untreated areas. It was recommended not to treat the same area with more than two steps. More steps could cause adverse effects, such as burns and hyperpigmentation. The application of anesthetics was not required due to the size of the treated areas and the laser’s depth of action, making this treatment easy and possible for all types of patients. Stretch mark changes were evaluated using the Manchester Scar Scale [40], and the mean scores related to each parameter at baseline, 3M FU and 6M FU after the last treatment session, were recorded. This scale comprises five parameters: contour, color, texture, distortion, and finish. All parameters range in a score scale of 1 to 4, except for finish, which was scored either 1 (matte) or 2 (shiny). These scores can be extended from a maximum of 18 points to a minimum of five points (lower scores indicate a better aesthetic appearance). Pain level was recorded using a Visual Analog Scale (VAS) of 5 points (0: none, 1: slight, 2, 3: moderate, 4: severe, 5: intolerable) to evaluate the patient’s comfort. Overall appearance assessment scores, from 0 to 4 (0: no improvement, 1: minimal improvement (<25%), 2: good improvement (25–50%), 3: very good improvement (50–75%), 4: excellent improvement (>75%)) were also recorded. Adverse events such as edema, crusting, scars, erythema, blisters, or dyschromia were evaluated at the post-treatment visit.

### 2.4. Post-Treatment Procedures

After the treatment, the patients refrigerated the area treated with cold gel, dried by dabbing, and locally applied a natural turmeric cream (HinoPro Solution Curcut, 200 mL), with a prolonged massage. Throughout treatment, patients are also recommended to use sun protection factors.

### 2.5. Statistical Analysis

Paired Student’s *t*-test was used to test all of the outcome data for statistical significance with the SPSS program version 25.0 (IBM, Armonk, NY, USA). The level of significance for statistical tests was (*p* < 0.01).

## 3. Results

**Figure 1:**
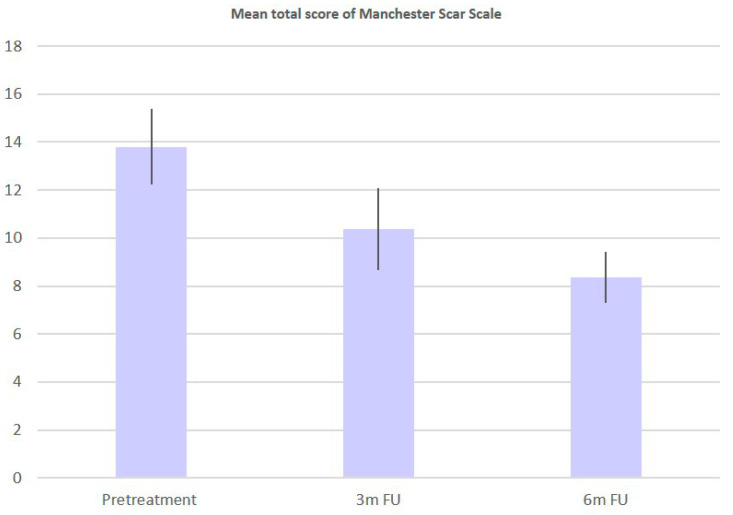
Histogram representation of the mean parameters scores of Manchester Scar Scale.

**Figure 2:**
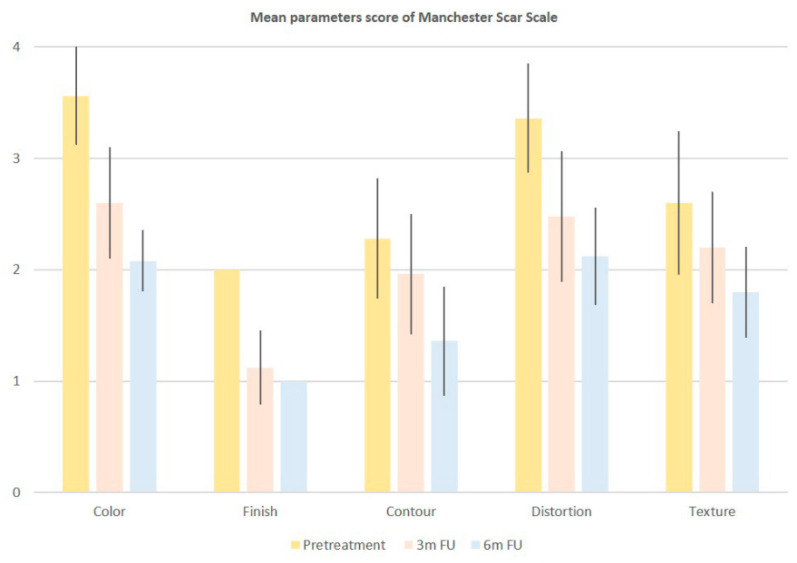
Histogram representation of the mean total score of Manchester Scar Scale.

**Figure 3:**
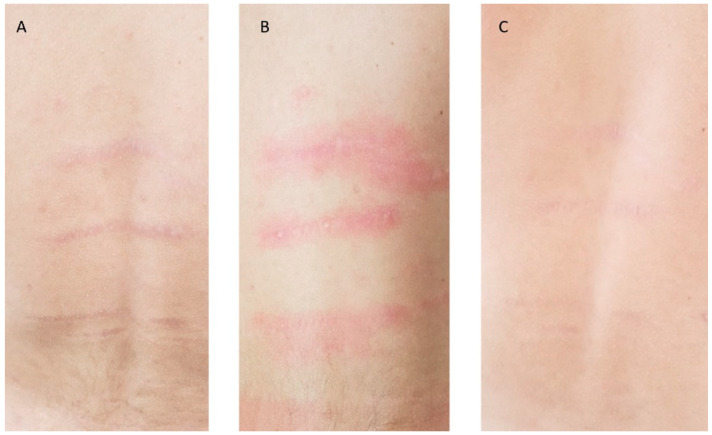
Photographic image of a male patient’s back area with Striae distensae (SD) before treatment (**A**), immediately after treatment (**B**), and 6M FU (**C**) after the last treatment with Nd:YAP 1340 nm laser. A visible and excellent aesthetic improvement of SD is shown.

**Figure 4:**
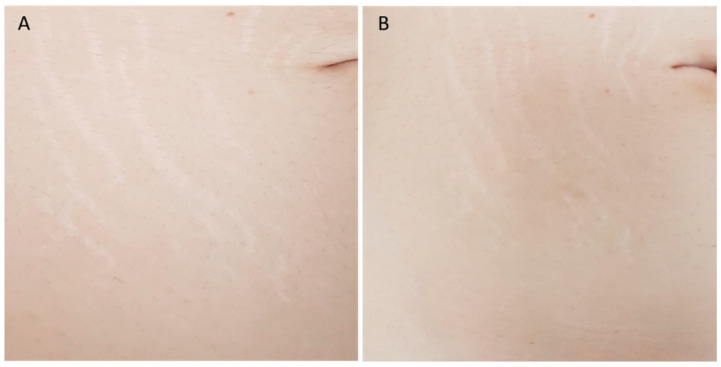
Photographic image of a patient’s umbilicus area with Striae distensae (SD) before treatment (**A**) and at 3M FU after the last treatment with Nd:YAP 1340 nm laser (**B**). A visible aesthetic improvement of SD is shown.

A total of 25 patients with a mean age of 31 ± 13.09 years participated in this study. The treated areas were the back, abdomen, flanks, breast, buttocks, and thighs/lower limbs. Mean scores related to each Manchester Scar Scale parameter, with their relative per-centage change, at baseline, 3M FU and 6M FU after the last treatment session were significantly improved and were reported in Table 1 and Figure 1. At 6-months post-treatment evaluation all patients displayed improvement of 41.57% in lesion col-or, 50.00% in lesion finish, 40.35% in lesion contour, 36.90% in lesion distortion and 30.80% in lesion texture, without long-lasting or severe adverse effects. The mean scores of finish (from baseline 2.00 ± 0.00 to 3M FU 1.12 ± 0.33 and to 6M FU 1.00 ± 0.00), Color (from baseline 3.56 ± 0.50 to 3M FU 2.60 ± 0.50 and to 6M FU 2.08 ± 0.28), Contour (from baseline 2.28 ± 0.54 to 3M FU 1.96 ± 0.53 and to 6M FU 1.36 ± 0.49), Distortion (from baseline 3.36 ± 0.49 to 3M FU 2.48 ± 0.58 and to 6M FU 2.12 ± 0.44) and Texture (from baseline 2.6 ± 0.64 to 3M FU 2.20 ± 0.50 and to 6M FU 1.80 ± 0.41) parameters were significantly decreased, showing excellent results (Table 1 and Figure 1). The total mean pretreatment Manchester Scar Scale score significantly decreased from 13.80 (±1.58) to 10.36 (±1.70) after 3 months (*p* < 0.01) and to 8.36 (±1.07) after 6 months (*p* < 0.01), (Table 2 and Figure 2). The digital photograph shows aesthetic results (see Figure 3 and Figure 4).

### 3.1. Pain Questionnaires

Treatment was well-tolerated by all patients (pain score, 1.08 ± 0.76). The pain was minimum for all subjects.

### 3.2. Overall Appearance Assessment

Overall striae improvement was evaluated and showed excellent improvement in 88% of patients and outstanding improvement in the remaining 12%. The mean score was 3.88 ± 0.33.

### 3.3. Side Effects

No long-term side effects were observed during the treatment period except slight redness and edema, both of which disappeared within 2/4 days.

## 4. Discussion

Although different therapeutic approaches are currently available, the treatment of stretch marks remains a challenge. Several and numerous treatments have been proposed, but with poor results [41]. For SD treatment, various laser parameters have been examined, alone or in combination with other treatment methods. Topical treatments have only mild effects and cannot prevent stretch marks in specific circumstances such as pregnancy [12,42,43,44,45,46]. As already mentioned in the introduction, many therapies are used for SD; the most recent is represented by laser and light therapies, particularly fractional lasers [36]. Over the years, excellent clinical results have been observed with non-ablative fractional lasers, which have become quite popular, particularly for their excellent tolerance and safety, even for patients with higher phototypes. Different non-ablative wavelengths, such as 1410, 1540, 1450, and 1565 nm, have been tested to treat SD [30,47]. The first fractional lasers introduced to the market were NAFLs, which can reach the dermis, leaving the epidermis intact [48].

In our study, already after three treatment sessions with the non-ablative fractional laser with a wavelength of 1340 nm, a positive effect could be observed, especially in adolescents. In this case, the technology advantage is that the laser beam, penetrating only 2–3 mm into the epidermis, induces minimal thermal effects. Our study results pointed to a SD improvement for all patients: significant amelioration of finish, texture, color, distortion, and contour, with a substantial increase in skin elasticity, was observed, and a remarkable improvement of SD in 88% of patients was revealed. It has also been observed that the 1340 laser, in combination with the ablative laser CO_2_, is effective in treating superficial scars. Further studies in this regard will be evaluated in the future to treat superficial stretch marks.

With no consumables, no need for topical anesthesia, and no preheating involved, the Nd:YAP 1340 nm (Insight Handpiece, Luxea, DEKA M.E.L.A., Calenzano, Italy) handpiece is easy to incorporate into any portfolio of treatment options. Its versatility allows the physician to treat various skin concerns, affording a larger patient cohort and increased patient satisfaction.

### Limitations of the Study

Limitations of the present study include the lack of histologic assessment and a limited number of patients.

## 5. Conclusions

The treatments available to ameliorate stretch marks’ appearance are far from optimal. Our study appears to demonstrate Nd:YAP 1340 nm laser efficacy, showing good tolerance for the treatment of more than one type of stretch mark placed in different body areas, with a significant improvement in skin texture/quality evaluated through the use of non-invasive-objective tools. This type of laser treatment is non-ablative and requires simple post-treatment management with a minimal and transitory limitation of the patient’s daily activities.

## Figures and Tables

**Table 1:** Parameters of Manchester Scar Scale and mean scores related to each parameter at 3M FU and 6M FU.

Parameters of Manchester Scar Scale N = 25	Baseline Mean ± SD	3M FU Mean ±SD	Percentage Change between Pre-Treatment and 3M FU	6M FU Mean ± SD	Percentage Change between Pre-Treatment and 6M FU
Color (Score range, 1–4)	3.56 ± 0.50	2.60 ± 0.50	26.96%	2.08 ± 0.28	41.57%
Finish (Score range, 1–2)	2.00 ± 0.00	1.12 ± 0.33	44.00%	1.00 ± 0.00	50.00%
Contour (Score range, 1–4)	2.28 ± 0.54	1.96 ± 0.53	14.03%	1.36 ± 0.49	40.35%
Distortion (Score range, 1–4)	3.36 ± 0.49	2.48 ± 0.58	26.19%	2.12 ± 0.44	36.90%
Texture (Score range, 1–4)	2.6 ± 0.64	2.20 ± 0.50	15.38%	1.80 ± 0.41	30.80%

**Table 2:** Total Manchester Scar Scale scores.

Manchester Scar Scale (Score Range, 1–18) N = 25	Mean ± SD	Percentage Change between Pre-Treatment and Follow Up (Significance)
Pre-treatment Score	13.80 ± 1.58	
3 Months Follow Up Score	10.36 ± 1.70	24.92% (*p* < 0.01)
6 Months Follow Up Score	8.36 ± 1.07	39.42% (*p* < 0.01)

## Data Availability

Data supporting this study findings are available on request from the corresponding author (IF).
